# 엄마 사랑해요

**DOI:** 10.4069/kjwhn.2020.11.13

**Published:** 2020-12-11

**Authors:** Eun Chae Park

**Affiliations:** Division of Nursing and Public Health, Daegu University, Gyeongsan, Korea; 대구대학교 간호보건학부

“엄마, 사랑해요”라는 말은 듣는 것도, 하는 것도 참 어려운 말 같다. 한 사람이 누군가를 만나고 결혼을 하여 새로운 생명이라는 엄청난 결실을 맺는다는 것은 이렇게 한 문장으로 끝맺을 수 있는 단순한 과정이 아니기 때문이다. 또, 성인이 되어 호기롭게 첫발을 내디딘 사회는 생각보다 너무 어둡고 차가워서 나를 아프게 하지만, 나에게 항상 따뜻한 엄마라는 존재는 나를 위해 다치게 하고 슬프게 해도 다 받아줄 것만 같아서 “사랑해요”라는 표현보다는 짜증을 내는 것이 점점 익숙해지기 때문이다.

나에게는 칠순을 바라보고 계시는 어머니와 사랑스러운 두 딸이 있다. 그리고 나는 간호사 생활을 한 지 어느덧 20년이라는 세월이 훌쩍 넘은 산부인과 간호사이다. 한 사람이 태어나고 자라 성인이 되는 세월을 간호사로서 후회 없이 보내왔지만, 힘든 순간이 없었던 것은 아니다. 처음 산부인과를 선택하게 된 이유는, 한 생명이 생겨나고 자라나 마침내 탄생하게 되는 전반적인 과정과 한 여성의 고유한 삶의 과정에 내가 잠시나마 함께 할 수 있으며 직∙간접적으로 영향을 미칠 수 있다는 것이 너무나 의미 있다는 생각이 들었고, 벅차올랐기 때문이다. 하지만 산부인과 간호사가 된다는 것은 그리 벅찬 순간만 있는 것은 아니었고, 탄생과 죽음을 모두 직접 마주해야 하는 힘든 일도 많았다. 그 어떤 순간에도 매사에 최선을 다하자고 생각했던 나는, 20년 세월 동안 딱 한 번 나의 직업에 회의를 느낀 적이 있었다.

하루는 환자들의 약물을 확인하고 있는 도중 갑자기 귀가 아플 정도의 목소리로 입에 담을 수 없는 욕설이 들려왔다. 그 순간 병동에 있던 모든 산모와 환자들, 간호사들이 얼어붙었고, 나는 급히 그 소리가 나오고 있는 병실로 향했다. 병실에 도착한 나는 차마 입을 다물 수가 없었다. 바닥에는 식판과 음식들이 널브러져 있고, 한 후배가 50대 정도 되어 보이는 남성에게 멱살을 잡혀 고개를 숙인 채 죄송하다고만 반복하고 있었기 때문이다. 순간 사고가 일시 정지되어 있다가, 이대로 두어서는 후배 혹은 다른 환자들이 다칠 수도 있겠다는 생각에 보호자를 말리기 시작했다. 다른 환자분들이 다치실 수도 있다며 진정하고 어떻게 된 건지 말씀해 주시면 가능한 선에서 빠르게 조처를 해드리겠다고 하자 그는 천천히 후배의 멱살을 놓았다. 나는 후배에게 나가서 잠시 쉬고 있으라 일러주고 보호자의 이야기를 들어 드렸다. 자신의 딸이 밥을 먹는 도중 머리카락이 들어 있는 것을 발견하고 간호사에게 바꿔 달라고 하였지만 30분이 지나도록 바꿔주지 않아 딸이 밥도 못 먹고 있는 상황이 화가 나서 그랬다는 것이다. 그날은 갑작스레 산모들과 환자들이 많이 입원한 날이라 종일 정신없이 일했던 날이었다. 물론 환자와 보호자의 입장은 충분히 이해가 되었지만, 바쁜 탓에 밥 한 끼 제대로 못 먹고 일했을 후배가 생각나 가슴이 무언가에 걸려 막힌 듯이 먹먹해져 왔다.

우선 보호자에게는 대신 죄송하다는 사과를 드리고 용서를 구한 후 곧바로 음식을 바꿔 드렸다. 그 후 급히 후배를 찾아가 보니, 뒤켠에 몰래 숨어서 울고 있던 후배는 내가 다가가자 눈물을 그치려고 최선을 다하며 나에게 계속 죄송하다고 했다. 괜찮다며 30분 동안 무엇을 하고 있었냐고 묻자, 갑자기 복통을 호소하는 환자가 있어 그 환자를 간호하고 있었다고 말하는 후배를 보고 나는 잠시 말을 꺼내지 못했다. 그리곤 다시 조심스레 그 환자는 어떻게 되었냐고 물으니 다행히 원인을 찾아내어 치료하고 안정되었다고 했다. 그 얘기를 듣고 나는 숨이 막혀왔다. 30분이라는 시간 동안 하나의 생명을 살리고 있었던 후배 간호사가 대견하기도 했고, 한편으론 미안한 마음도 들었다. 드라마에서만 벌어질 듯한 이야기가 실제 내 주변에서, 나와 가까운 사람에게서 일어났다는 사실이 두렵고 괴롭기도 했다. 누군가의 귀한 딸로 태어나 항상 사랑받아 마땅해야 할 이 아이가, 자기보단 남을 위해 희생하며 한 생명을 살리면서도 다른 누군가의 부모님, 그리고 다른 누군가의 자녀로부터 치욕스러운 모욕을 묵묵히 들어야만 하는 현실이, 몰래 혼자 눈물을 훔치며 연신 죄송하다고 말할 수밖에 없는 이 고통스러운 현실이 너무나 안타까워 나는 차마 아무 말도 하지 못하고 그저 그 아이의 손을 잡아줄 수밖에 없었다.

삶은 탄생(‘B’irth)과 죽음(‘D’eath)이고 그 사이에 선택(‘C’hoice)이 있다는 말이 있을 정도로 우리는 매일 여러 번의 선택을 한다. 그리고 우리는 그 선택의 주체로서, 결과가 좋든 나쁘든 책임을 지게 된다. 3년 전 그 일은 나에게 ‘선택’의 책임에 대해 한 번 더 생각하게 했다. 추운 겨울, 오전 근무를 위해 새벽에 준비를 마친 후 딸들이 자고 있는 방에 조용히 들어가 볼에 뽀뽀를 하고 일어서서 방을 나가려고 하자 중학생인 큰 딸이 눈을 비비며 일어나 “엄마, 사랑해요. 오늘도 파이팅!”이라고 말을 해주었다. 그 모습이 너무나 예쁘면서도, 항상 잘 챙겨주지 못해 미안한 마음에 꼭 껴안고 “엄마도 사랑해” 라는 말을 전한 뒤 집을 나섰다. 그리곤 병원까지 걸어가면서 방금 있었던 일을 떠올리다 문득 엄마 생각이 나 엄마께 전화를 드렸다. 이른 시간임에도 거의 바로 전화를 받으신 엄마의 목소리가 평소와 사뭇 달라 무슨 일이라도 있는지 여쭙자, 감기에 걸렸다고 하셨다. 일찍이 아버지가 돌아가신 탓에 혼자서 생활하시는 엄마를 평소에 신경 써드리지 못한 것이 죄송하고 울컥해서, 아프시면 참지 말고 나한테 이야기 좀 하라며 오히려 괜한 짜증을 냈다. 그러고는 병원에 들어가 봐야 한다며 나중에 연락하겠다고 급히 전화를 끊어버렸다.

그렇게 가슴 한 켠에 무거운 마음을 안고 평소처럼 환자와 산모에게 필요한 소독을 하던 중 유난히 눈에 띄는 한 사람, 딱 봐도 중고등학생쯤 되어 보이는 여자아이가 혼자서 배가 아프다며 찾아왔다. 병동의 모든 이목은 그 아이에게 집중되어 여기저기서 수군거리는 소리가 들리기 시작했다. 그런 시선 탓인지 아이의 몸은 떨리고 있었고, 무섭고 두려워 보였다. 하던 일을 마치고 동료 간호사의 부름에 아이의 초음파 결과지와 입원 사유를 보게 된 나는 당황하여 말을 얼버무릴 수밖에 없었다. 아이의 뱃속에는 한 생명체가 살아 숨 쉬고 있었기 때문이다. 알고 보니 아이는 미혼모로, 조기진통으로 인해 배가 아픈 것이었고, 안정을 위한 노력에도 불구하고 결국 며칠 후 응급으로 분만을 하게 되었다. 다행히 아이와 태중의 아기 모두 건강했고 미숙아로 태어난 아기는 인큐베이터로, 여자아이는 병실로 가게 되었다. 그 후 아무 일 없이 평소처럼 지나간 다음 날, 갑자기 후배 간호사가 달려오며 다급한 목소리로 “어제 응급으로 분만한 환자 없어졌어요!”라고 소리치자 나는 머릿속이 새하얘졌다. 다시 정신을 차리고 다른 과에 전화를 돌리며 그 아이를 찾기 위해 병원 이곳저곳을 뛰어다녔지만, 결국 찾을 수 없었다. 순간 다리 힘이 풀린 나는 비상계단에 앉아 한참을 아무것도 할 수 없었다. 나의 큰딸과 비슷한 나이의 그 여리고 작았던 아이는 얼마나 무섭고 두려웠을까, 태어난 아기는 앞으로 어떤 삶을 살아가게 될까. 내가 감히 가늠할 수조차 없는 앞으로의 일들에 대한 생각이 나의 머릿속을 온통 뒤덮어 놓았다. 결국 태어난 아기는 보육 시설로 보내졌고, 나는 며칠 동안 그 아이에 대한 생각의 끈을 놓을 수 없었으며, 그런 ‘선택’을 하게 만들었을 이 사회의 눈초리가 미웠다.

그렇게 며칠을 잠도 잘 못 자고 힘들어하다 보니 불현듯 엄마 생각이 났고, 지난번 엄마에게 짜증을 냈던 일로 미안한 마음에 약간 망설이다가 엄마에게 전화를 걸었다. 병원 일에 치여서 바쁘다는 핑계로 이제껏 제대로 효도 한 번 못한 딸의 전화를 엄마는 마치 기다렸다는 듯이 받으셨다. 밥은 먹었냐며 내 안부를 먼저 생각하시는 말씀에 나는 몇 분 동안 아무 말 못 하고 눈물만 흘렸다. 그런 딸의 전화를 묵묵히 받아 주시던 엄마께 언제 해본 지도 기억에 없는 “엄마, 사랑해요”라는 말을 전했을 때, 약간의 침묵 뒤 “엄마도 사랑한다. 하나밖에 없는 우리 딸, 항상 고맙고 미안해”라고 말씀하시던 떨리는 엄마의 음성은 여전히 내 가슴 속 깊이 남아 있다.

우리는 모두 누군가에게 소중한 딸이고 아들이다. 하지만 간호복을 입은 후 간호사들은 나를 위해서가 아닌 다른 사람들을 위해 자신을 희생하고 최선을 다하고 있다. 부디 간호사들을 존중해주고 그들의 노력과 헌신을 가볍게 여기지 말아주기를, 그리고 나이와 상관없이 모든 여성들이 산부인과 진료를 맘 편히 받을 수 있게 따뜻한 눈으로 바라봐 주기를 간절히 바란다. 비록 작은 힘일지라도 나는 모든 여성이 남의 눈을 신경 쓰지 않고 적절하게 간호받을 수 있도록 노력할 것이며, 어떤 상황이 다가와도 언제, 어디서든 환자가 있는 곳이라면 기꺼이 달려갈 것이다.

